# Increased CSN5 expression enhances the sensitivity to lenalidomide in multiple myeloma cells

**DOI:** 10.1016/j.isci.2024.111399

**Published:** 2024-11-15

**Authors:** Takumi Yamamoto, Arisu Furukawa, Yue Zhou, Nobuaki Kono, Shojiro Kitajima, Hiroto Ohguchi, Yawara Kawano, Shingo Ito, Norie Araki, Sumio Ohtsuki, Takeshi Masuda

**Affiliations:** 1 Department of Pharmaceutical Microbiology, Graduate School of Pharmaceutical Sciences, Kumamoto University, Kumamoto 862-0973, Japan; 2 Department of Cancer Cell Biology, Faculty of Pharmaceutical Sciences, University of Toyama, Toyama 930-0194, Japan; 3 Institute for Advanced Biosciences, Keio University, Yamagata 997-0017, Japan; 4 Division of Disease Epigenetics, Institute of Resource Development and Analysis, Kumamoto University, Kumamoto 860-0811, Japan; 5 Department of Hematology, Rheumatology, and Infectious Disease, Faculty of Life Sciences, Kumamoto University, Kumamoto 860-8556, Japan; 6 Department of Pharmaceutical Microbiology, Faculty of Life Sciences, Kumamoto University, Kumamoto 862-0973, Japan; 7 Department of Tumor Genetics and Biology, Graduate School of Medical Sciences, Kumamoto University, Kumamoto 860-8556, Japan

**Keywords:** Molecular biology, Cell biology, Cancer, Proteomics

## Abstract

Lenalidomide (LEN) is commonly used as an effective therapeutic agent for multiple myeloma (MM). However, in some patients, primary resistance to LEN is observed, the mechanisms of which remain poorly understood. In this study, we combined a LEN sensitivity assay with proteomics data from 15 MM cell lines to identify protein expression profiles associated with primary LEN resistance. Our findings revealed that CSN5 expression is lower in LEN-resistant cell lines than in LEN-sensitive lines. Moreover, we established that CSN5 is degraded via the cullin-RING ubiquitin ligase (CRL)-mediated ubiquitin-proteasome pathway through ubiquitination at lysine 194. Our data suggest that reduced CSN5 expression leads to abnormalities in the ubiquitination cycle of CRL4A, resulting in the inhibition of LEN-mediated degradation of IKZF1 and IKZF3. These findings delineate an additional mechanism of LEN resistance in MM cells and may contribute to the development of alternative therapeutic strategies to overcome LEN resistance.

## Highlights

• CSN5 expression is lower in LEN-resistant cell lines than in LEN-sensitive lines.
• Overexpression of CSN5 in multiple myeloma cell lines increased sensitivity to LEN.
• CSN5 degradation via the ubiquitin-proteasome pathway is enhanced in LEN-resistant cells.
• Downregulation of CSN5 disrupts ubiquitination cycle of CRL4A.

## Graphical abstract

**Figure:**
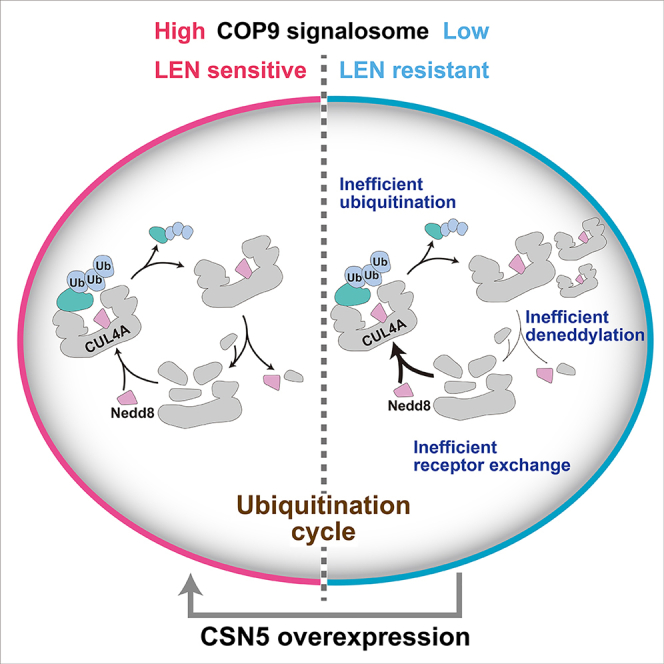

## Introduction

Lenalidomide (LEN) is used to treat multiple myeloma (MM)^1^ and exerts its therapeutic efficacy through diverse effects, including antitumor, anti-angiogenic, and anti-inflammatory activity and the activation of natural killer cells.^2^^,^^3^^,^^4^ In terms of its antitumor activity, LEN acts as a molecular glue to recruit the neo-substrates Ikaros family zinc finger (IKZF)1 and IKZF3 to cereblon (CRBN), a substrate receptor of the cullin 4A-RING E3 ubiquitin ligase.^5^ The ubiquitination and subsequent proteasomal degradation of IKZF1 and IKZF3 lead to the downregulation of transcription factors IRF4 and c-Myc,^6^^,^^7^^,^^8^ which are crucial for the survival of MM cells.^9^

E3 ubiquitin ligases are categorized into four major types, and cullin 4A belongs to the largest family, known as cullin-RING E3 ubiquitin ligases (CRLs).^10^^,^^11^^,^^12^ CRLs are multisubunit complexes consisting of cullin, the RING domain protein Rbx1, an adaptor protein, and a substrate receptor protein. CRLs are activated by Nedd8 modification (neddylation) of cullin and inactivated by Nedd8 removal (deneddylation). Deneddylation is carried out by the COP9 signalosome complex.^13^ After deneddylation, Cand1 disassembles the adaptor and substrate receptor proteins by interacting with cullin^14^ and is released when the adaptor and substrate receptors are re-recruited. Subsequently, cullin is activated by neddylation through DCN-mediated interaction with UBC12. This ubiquitination cycle driven by neddylation enables CRLs to ubiquitinate the next substrate.

Approximately 40% of patients with MM have primary resistance to LEN.^15^ Furthermore, even in patients who are initially responsive, acquired resistance is induced after prolonged use. Although the mechanism of action of LEN and the regulatory mechanism of CRL activity have been largely clarified as described previously, the mechanisms of primary resistance to LEN in MM cells are not fully understood. Tremendous efforts have been made to elucidate these mechanisms. Large-scale genome and exon sequencing studies of MM patients have revealed that only 10%–20% of LEN-resistant patients have mutations in CRBN axis-related genes, such as *CRBN*, *IKZF1*, and *IRF4*.^16^^,^^17^ In addition, numerous genetic mutations in MAPK pathway genes and *TP53* have been observed in LEN-resistant patients with MM^16^, yet only a limited number of these mutations contribute to LEN resistance.^18^^,^^19^ Genome-scale CRISPR screens to identify novel genes involved in LEN sensitivity have led to the identification of not only known genes involved in the CRBN axis, but also ubiquitination and neddylation regulators, and have advanced our understanding of the mechanism of action of LEN.^19^^,^^20^^,^^21^ However, expression changes in the genes identified by CRISPR screening have not been observed in clinical specimens of LEN-resistant patients.^18^^,^^19^ In addition, post-translational modifications, including ubiquitination and neddylation, as well as protein degradation are involved in the CRBN axis. Therefore, it is important to assess the mechanism of primary LEN resistance at the protein level rather than at the gene level.

In this study, we aimed to elucidate the mechanisms involved in primary resistance to LEN. It is expected that an understanding of the mechanisms of primary resistance would allow early prediction and diagnosis, enabling personalized treatment plans and higher success rates. Also, it may be possible to avoid unnecessary side effects and reduce costs by preventing ineffective treatments. Furthermore, it aids in devising preventive strategies and improves the design of clinical trials, leading to more accurate evaluation of effective therapies. In this study, we performed a combination analysis involving a LEN sensitivity assay and large-scale proteomics data from 15 MM cell lines, which were not treated with LEN, to assess expression changes in endogenous proteins associated with primary resistance to LEN. We found that the endogenous expression level of CSN5, a component of the COP9 signalosome responsible for deneddylation, was decreased in LEN-resistant cells, and that enhancing CSN5 expression resulted in increased sensitivity to LEN. Based on these results, we propose an LEN resistance mechanism involving an aberration in the ubiquitination cycle caused by a decrease in deneddylation activity. These findings provide a potential therapeutic target to overcome primary resistance to LEN.

## Results

### COP9 signalosome components are downregulated in LEN-resistant cell lines

**Figure 1:**
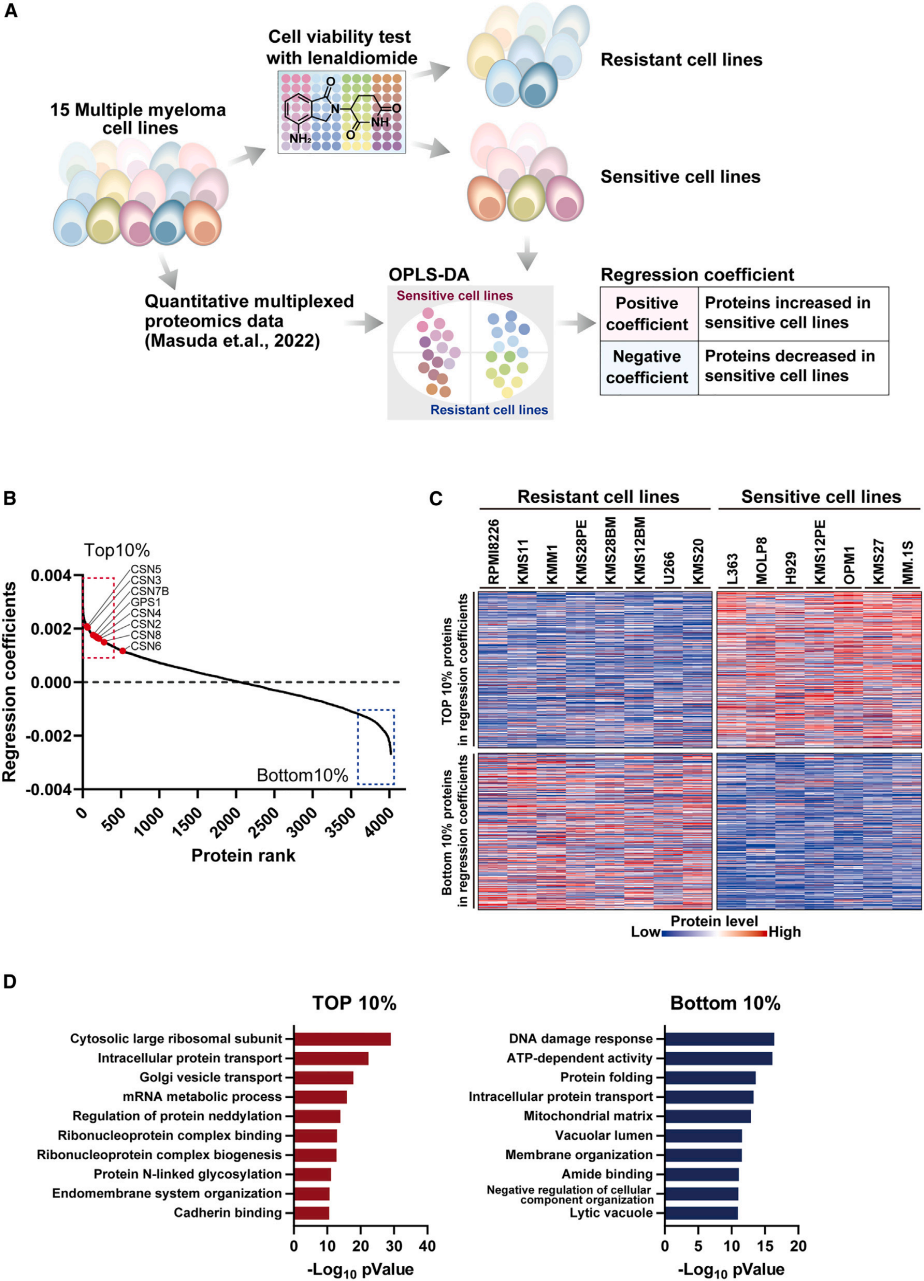
Identification of the proteins involved in LEN resistance (A) Workflow used for the identification of proteins involved in LEN resistance. Based on LEN sensitivity assay results, the 15 MM cell lines were classified into sensitive and resistant groups. The proteomics data of each cell line were used for OPLS-DA in SIMCA 17.0 to differentiate between the LEN-sensitive and -resistant groups, yielding regression coefficients for all proteins. (B) Regression coefficients for the proteins identified. (C) Expression profiles of the top 10% and bottom 10% proteins based on regression coefficients. Red and blue indicate high and low protein expression, respectively. (D) GO enrichment analysis of the top 10% and bottom 10% proteins based on the regression coefficients using Metascape (https://metascape.org/).

To identify the characteristics of proteome profiles in cells showing primary resistance to LEN, we first conducted LEN-sensitivity assays using 15 MM cell lines, which were not treated with LEN (Figure 1A). LEN sensitivity was defined as the ratio of cell viability in the presence of 100 μM LEN to that in a DMSO control (Figure S1A). Based on the assay results, the 15 cell lines were classified into two groups: a LEN-resistant group (RPMI8226, KMS11, KMM1, KMS28PE, KMS28BM, KMS12BM, U266, and KMS20) and a LEN-sensitive group (L363, MOLP8, H929, KMS12PE, OPM1, KMS27, and MM.1S). Published protein expression data for each cell line (*n* = 3)^22^^,^^23^ were reanalyzed. Out of 6807 proteins quantified, 4020 proteins were quantified in more than half of the samples and were used to compare their expression levels in the LEN-sensitive and -resistant groups, to determine their expression levels in each cell line (Table S1), and to assess the correlation between their expression levels and cell viability. The data are available in MM Proteome Data (https://mmproteomicsdata.iab.keio.ac.jp/). We found no significant difference in CRBN expression between the LEN-sensitive and -resistant groups (*p* = 0.852) and no significant correlation between its expression level and LEN sensitivity (*p* = 0.698) (Figure S1B). We also evaluated the mutation profiles of the LEN-resistant cell lines (RPMI8226, KMS11, KMM1, KMS28PE, KMS28BM, KMS12BM, U266, and KMS20) using the Depmap portal (https://depmap.org/portal/). No mutation in CRBN, IKZF1/3, and IRF4 was detected in any of these cell lines. These data indicated that LEN sensitivity in the cell lines used in this study does not depend on CRBN expression or on the mutation of the CRBN-axis-related genes.

Next, we performed an orthogonal partial least squares discriminant analysis (OPLS-DA) of the LEN-sensitive and -resistant cell groups using the 4020 proteins (Figure S1C). Regression coefficients representing the differences in protein expression levels between the resistant and sensitive cell lines were obtained. Positive coefficients indicated higher expression in sensitive cells, whereas negative coefficients indicated higher expression in resistant cells. We focused on the top and bottom 10% of proteins with the highest and lowest regression coefficients (Figure 1B). The selected proteins showed expression patterns consistent with the expectations, showing higher expression in either the sensitive or the resistant cell groups (Figure 1C). A functional enrichment analysis was performed using the selected proteins (Figure 1D). In the protein group with higher expression in the sensitive cell lines (Top 10% of proteins), proteins related to the cytosolic large ribosomal subunit were the most significantly enriched. In the mRNA metabolic process, including enhancer of mRNA-decapping protein 4 (EDC4), was the fourth most significantly enriched group. In addition, regulators of protein neddylation, which are reportedly involved in the CRBN axis, were identified. In the protein group with lower expression in sensitive cells (Bottom 10% of proteins), DNA damage response-related proteins, including ribonucleoside-diphosphate reductase large subunit (RRM1), were the most significantly enriched.

**Figure 2:**
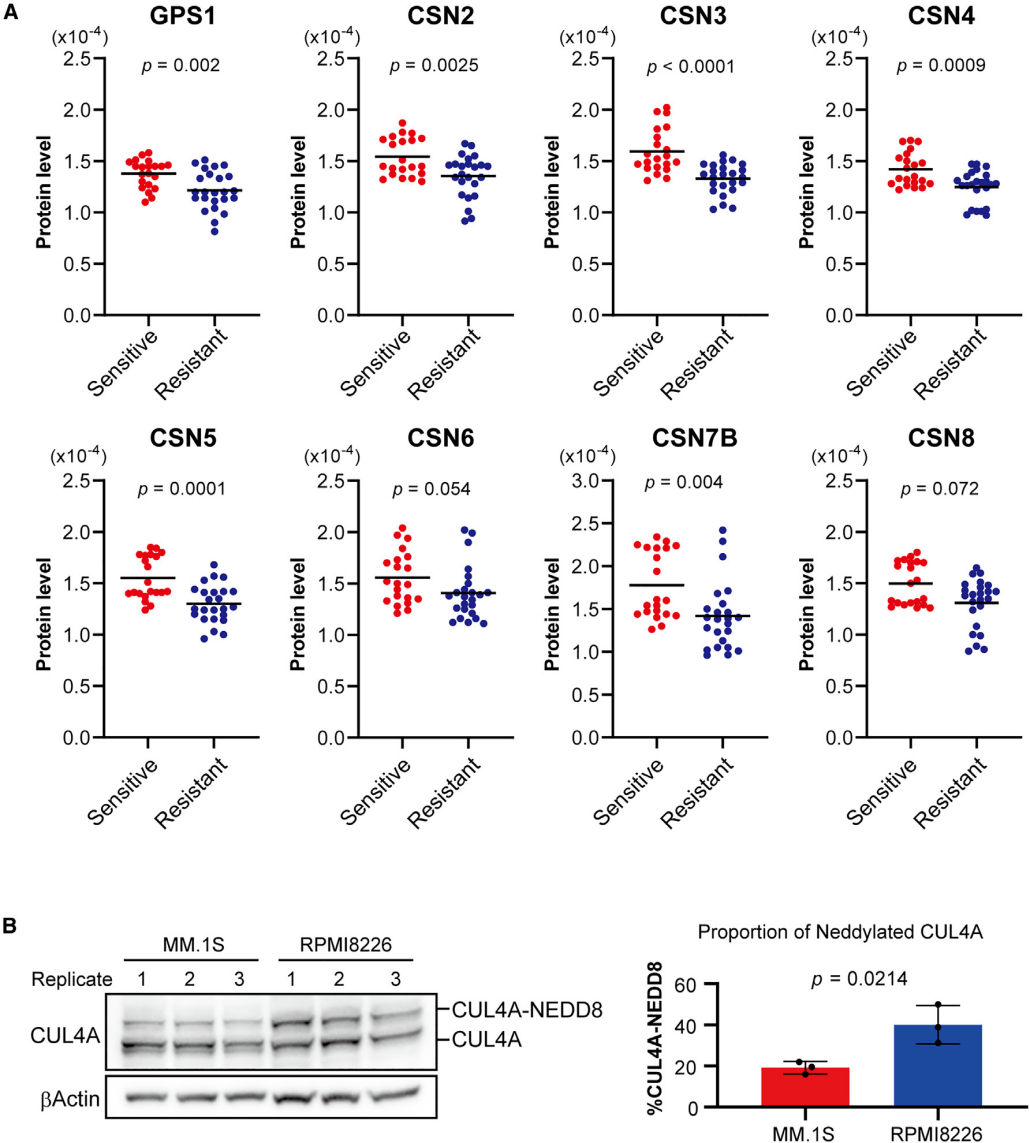
COP9 signalosome protein expression in LEN-sensitive and -resistant groups (A) Comparison of the expression levels of COP9 signalosome proteins identified through proteomics. Student's t test. (B) Comparison of the proportions of neddylated- and non-neddylated-CUL4 in MM.1S and RPMI8226 cells, which were the most LEN-sensitive and -resistant cells, respectively, among the 15 cell lines assessed in this study. Comparisons were performed in triplicate. Band densities were quantified using iBright Analysis Software, and were averaged across the data obtained for triplicate assessments. Error bar represents standard deviation. Student's t test.

Based on the previous results, we focused our subsequent analyses on the proteins associated with the regulation of protein neddylation. The ubiquitination activity of the CRL complex is activated by neddylation of cullin and inactivated by deneddylation mediated by the COP9 signalosome,^22^ suggesting that the expression levels of neddylation-related proteins may affect LEN sensitivity. Among the proteins related to the regulation of protein neddylation, we focused on the COP9 signalosome, which consists of eight proteins (Figure S2). Importantly, the proteins with top-10% regression coefficients in OPLS-DA included COP9 signalosome components, other than CSN6, among which CSN5 had the highest regression coefficient (Figure 1B). The expression levels of GPS1 (*p* = 0.002), CSN2 (*p* = 0.0025), CSN3 (*p* < 0.0001), CSN4 (*p* = 0.0009), CSN5 (*p* = 0.0001), and CSN7B (*p* = 0.004) were significantly decreased in LEN-resistant cell lines compared to LEN-sensitive cell lines (Figure 2A). Although there were no significant differences in the expression levels of CSN6 (*p* = 0.054) and CSN8 (*p* = 0.072), these proteins tended to be decreased in expression in the resistant cell lines. The proteins other than CSN6 were all included in the top 10% of proteins with the highest regression coefficients. Next, to evaluate whether the reduced expression of COP9 signalosome components affected deneddylation activity, we compared the amounts of neddylated and unneddylated CUL4A in MM.1S and RPMI8226 cells, which showed the highest LEN sensitivity and resistance, respectively. The proportion of neddylated CUL4A was significantly 2.1-fold higher in the RPMI8226 cells than in the MM.1S cells (*p* = 0.0214) (Figure 2B), suggesting that the decreases in the expression levels of COP9 signalosome components were associated with deneddylation activity.

### Overexpression of CSN5 induces LEN sensitivity

**Figure 3:**
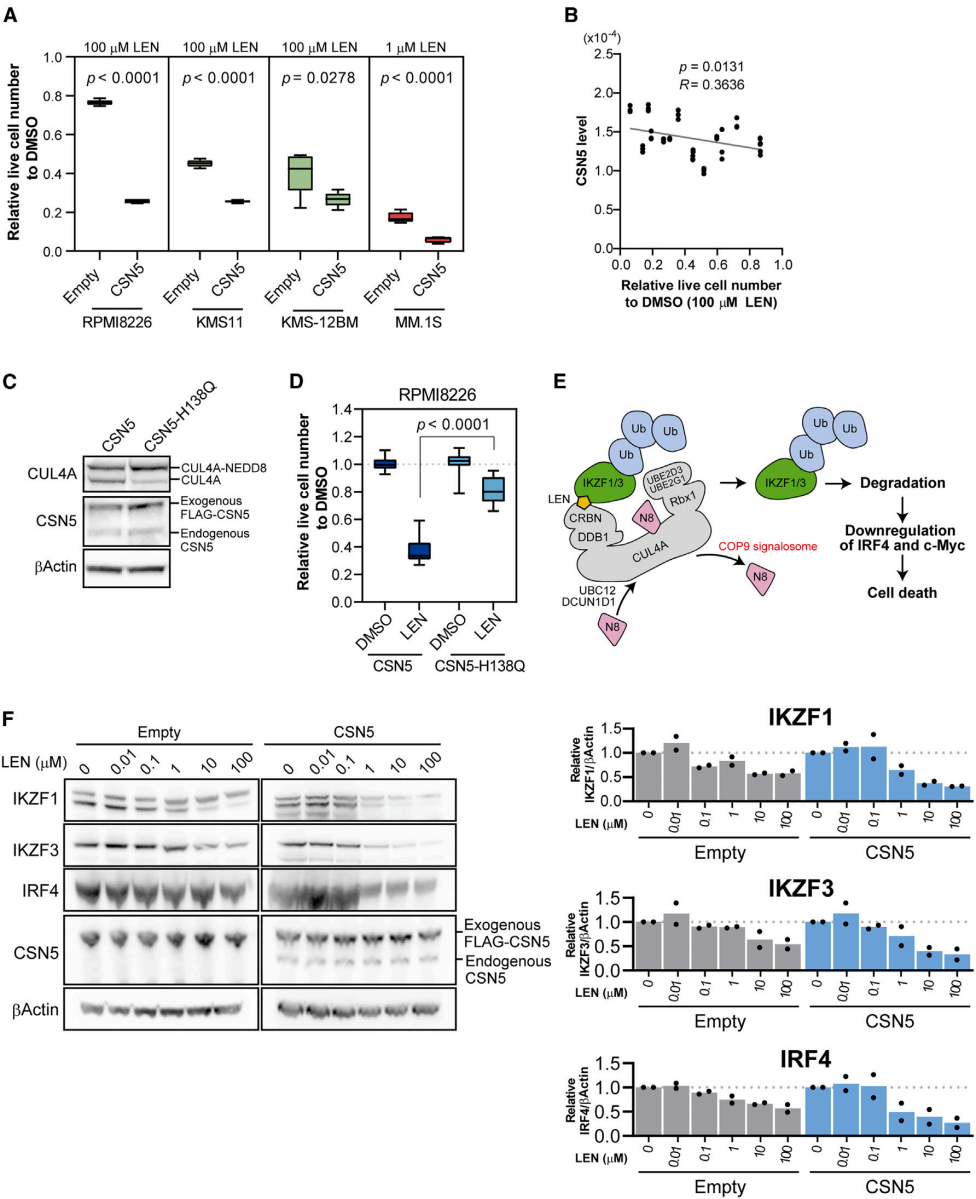
Overexpression of CSN5 induces LEN sensitivity (A) Induction of LEN sensitivity in RPMI8226, KMS11, KMS12BM, and MM.1S cells overexpressing FLAG-CSN5. Empty vector (lentiEF1-FLAG-P2A-Blast) was used as a control. We used 100 μM and 1 μM LEN for LEN-resistant and -sensitive cells, respectively. Data represent the mean ± standard deviation (*n* = 5). Student's t test. (B) Relationship between LEN sensitivity and the endogenous CSN5 expression levels in the 15 cell lines. Correlations between protein levels and cell viability were evaluated using simple linear regression in GraphPad Prism 8. Statistical significance was set at *p* < 0.05. (C) Western blots representing the expression of CUL4A and CSN5 in RPMI8226 cells overexpressing WT FLAG-CSN5 or FLAG-CSN5-H138Q. β-actin was used as a loading control. The larger proportion of neddylated CUL4A suggested that CSN5-H138Q decreased deneddylation activity. (D) LEN sensitivity in RPMI8226 cells overexpressing FLAG-CSN5-H138Q. The lentiEF1-FLAG-P2A-Blast vector harboring FLAG-tagged CSN5 or FLAG-tagged CSN5 with H138Q mutation was used for lentiviral infection with RPMI8226. We used 100 μM LEN in this assay. Data represent the mean ± standard deviation (*n* = 5). Student's t test. (E) LEN response pathway (CRBN axis) involved in the antitumor mechanism of LEN. (F) Western blots of IKZF1, IKFZ3, IRF4, CSN5 expression levels after treatment with the indicated dose of LEN in FLAG-CSN5- or empty vector-overexpressing RPMI8226 cells. β-Αctin was used as the loading control. The protein bands in western blotting of IKZF1, IKZF3 and IRF4 were quantified using iBright Analysis Software. Band densities of IKZF1, IKZF3 and IRF4 were normalized to that of β-actin. Data represent the mean (*n* = 2).

The knockdown or knockout of COP9 signalosome components has been demonstrated to induce LEN resistance.^20^^,^^21^ We investigated whether increasing COP9 signalosome expression and enhancing deneddylation activity could induce LEN sensitivity. Because it is difficult to simultaneously overexpress all eight COP9 signalosome components, only CSN5 was overexpressed in this study as it is the component responsible enzyme for deneddylation activity^23^ and also has the highest regression coefficient in OPLS-DA among the COP9 signalosome components as shown in Figure 1B. Mutations in CSN5 are known to result in the loss of deneddylation activity of the COP9 signalosome.^24^^,^^25^ We overexpressed CSN5 in three LEN-resistant cell lines (RPMI8226, KMS11, and KMS12BM) and one LEN-sensitive cell line (MM.1S) (Figure S3A) and evaluated their sensitivity to 100 μM and 1 μM LEN, respectively. In all resistant cell lines, relative live cell numbers were down to approximately 0.2 in the presence of 100 μM LEN by CSN5 overexpression (Figure 3A). Interestingly, the sensitivity to LEN was also enhanced in the LEN-sensitive cell line (MM.1S) overexpressing CSN5 (Figure 3A). In RPMI8226 cells overexpressing CSN5, cell viability decreased in a concentration-dependent manner following LEN treatment (Figure S3B). Moreover, a significant negative correlation (*p* = 0.0131) between CSN5 levels and cell viability in the presence of LEN was found in the 15 MM cell lines (Figure 3B).

Next, to investigate the requirement of deneddylation activity for the induction of LEN sensitivity by CSN5, we established RPMI8226 cells overexpressing CSN5-H138Q (Figure 3C). The histidine at position 138 reportedly is crucial for deneddylation, and its conversion to glutamine or alanine leads to the loss of deneddylation activity.^23^^,^^26^^,^^27^^,^^28^ In RPMI8226 cells overexpressing CSN5-H138Q, LEN sensitivity was not induced (Figure 3D), suggesting that deneddylation activity is necessary for the induction of LEN sensitivity. Deneddylation is carried out by the entire COP9 signalosome complex, not CSN5 alone.^29^ Interestingly, overexpression of only CSN5 increased the proportion of unneddylated CUL4A (Figure S3C). We hypothesized that CSN5 overexpression induced the expression of other COP9 signalosome components. To examine this hypothesis, we performed a proteomics analysis using RPMI8226 cells overexpressing CSN5. CSN5 level was significantly increased more than 2-fold in RPMI8226 overexpressing CSN5, as expected (*p* < 0.001) (Figure S3D). GPS1 (*p* = 0.006), CSN2 (*p* = 0.002), CSN3 (*p* = 0.048), CSN4 (*p* = 0.016), CSN7A (*p* < 0.001), and CSN7B (*p* < 0.001) also showed significant changes in expression (Figure S3D). However, these changes were within 38%.

Overexpression of CSN5 inhibited the growth of the MM cell lines upon LEN treatment. However, the underlying mechanism remained unknown. To examine whether the inhibition of cell proliferation was induced via the CRBN axis (Figure 3E), we observed the degradation of IKZF1 and IKZF3 and the subsequent decrease in IRF4 expression upon LEN treatment. IKZF1 and IKZF3 degradation was enhanced in the presence of 1 μM LEN in RPMI8226 cells overexpressing CSN5 compared to control RPMI8226 cells (Figure 3F). Furthermore, increasing the concentration of LEN led to a corresponding increase in the extent of IKZF1 and IKZF3 degradation. Additionally, IRF4 expression decreased in accordance with the amount of IKZF1 and IKZF3. Similar results were obtained with another LEN-resistant cell line, KMS12BM (Figure S3E). These findings indicated that the CRBN axis, which was less active in control RPMI8226 and KMS12BM cells, was enhanced upon CSN5 overexpression.

### CSN5 degradation via the ubiquitin-proteasome pathway is enhanced in LEN-resistant cells

**Figure 4:**
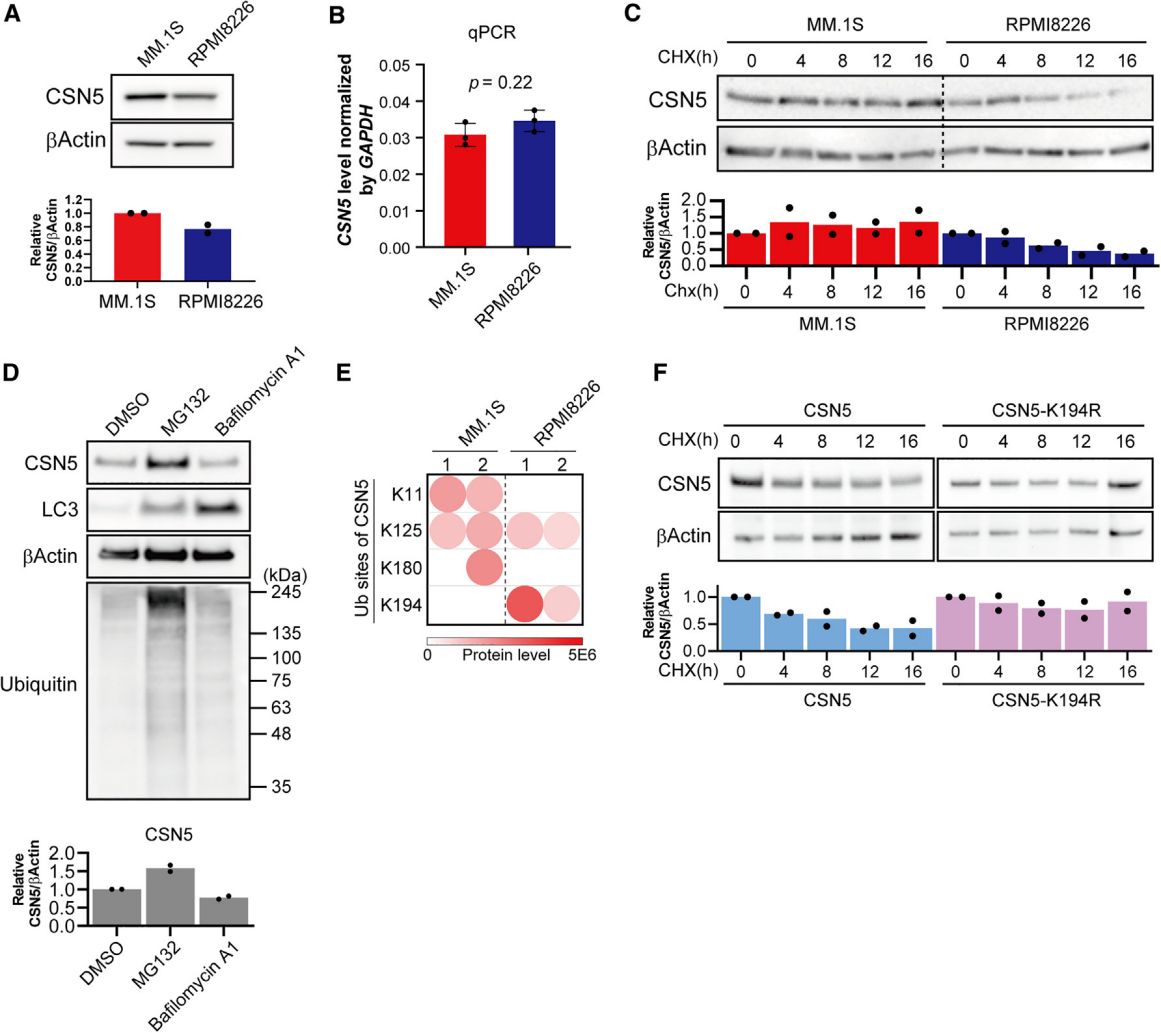
Mechanism underlying the low expression of CSN5 in LEN-resistant cells (A) CSN5 protein level was compared in MM.1S and RPMI8226 cells. Data represent the mean (*n* = 2). (B) CSN5 mRNA level was compared in MM.1S and RPMI8226 cells. Data represent the mean ± standard deviation (*n* = 3). Student's t test. (C) Comparison of CSN5 degradation time between MM.1S and RPMI8226 cells using a CHX chase assay. The cells were incubated with 10 μM CHX. Data represent the mean (*n* = 2). (D) Western blots of CSN5, LC3, and ubiquitin levels after treatment with the proteasome inhibitor MG132 or the autophagy inhibitor bafilomycin A1 in RPMI8226 cells. β-Αctin was used as the loading control. Data represent the mean (*n* = 2). (E) Identification of ubiquitination sites on CSN5 in MM.1S and RPMI8226 cells by proteomics. The experiment was conducted in technical duplicate. (F) Comparison of CSN5 degradation time in RPMI8226 cells overexpressing FLAG-CSN5 or CSN5-K194R using a CHX chase assay. The cells were incubated with 10 μM CHX. Band densities were quantified using iBright Analysis Software. Data represent the mean (*n* = 2).

To investigate the mechanisms underlying the decrease in endogenous CSN5 expression in LEN-resistant cell lines, we used MM.1S and RPMI8226 cells. Western blot analysis of CSN5 levels revealed lower expression of CSN5 in RPMI8226 cells than in MM.1S cells (Figure 4A). This finding was consistent with the proteomics analysis results. We considered that the decrease in CSN5 expression may have been due to a reduction in gene transcription or an increase in protein degradation. First, we assessed *CSN5* mRNA expression using quantitative PCR (qPCR). We found no significant difference in *CSN5* mRNA levels between MM.1S and RPMI8226 cells (Figure 4B, *p* = 0.22). Next, we compared CSN5 degradation using a cycloheximide (CHX) chase assay. After 16 h of CHX treatment, the amount of CSN5 in MM.1S cells remained similar to that at the 0 h, whereas CSN5 levels in RPMI8226 cells began to decrease after 8 h (Figure 4C), indicating a faster CSN5 degradation rate in RPMI8226 cells than in MM.1S cells. We next investigated CSN5 degradation, focusing on the two major pathways, the proteasome and autophagy, using MG132 and bafilomycin A1 to inhibit the respective pathways (Figure 4D). CSN5 accumulated upon the addition of MG132, but not bafilomycin A1, suggesting that CSN5 is degraded via the ubiquitin proteasome pathway.

To identify the ubiquitination sites involved in CSN5 degradation, we conducted a ubiquitin proteome analysis, which identified that the lysine residues at positions 11, 125, and 180 of CSN5 were ubiquitinated in MM.1S cells, whereas those at positions 125 and 194 were ubiquitinated in RPMI8226 cells (Figure 4E). The lysine residue at position 194 (K194) was a unique ubiquitination site in RPMI8226 cells. Moreover, the amount of peptides ubiquitinated at K194 was higher in LEN-resistant KMS27 cells than that in LEN-sensitive KMS11 cells (Figure S4). Protein ubiquitination is involved in signaling as well as proteasomal degradation.^30^^,^^31^ To investigate whether K194 ubiquitination is involved in the degradation of CSN5, we performed a CHX chase assay using RPMI8226 cells expressing CSN5-K194R. The wild-type CSN5 level decreased to 42%, 16 h after the addition of CHX, whereas the level of CSN5-K194R decreased to only 92% (Figure 4F). These data suggested that CSN5 degradation was accelerated by K194 ubiquitination in the resistant RPMI8226 cells.

### CSN5 is ubiquitinated by CRL

**Figure 5:**
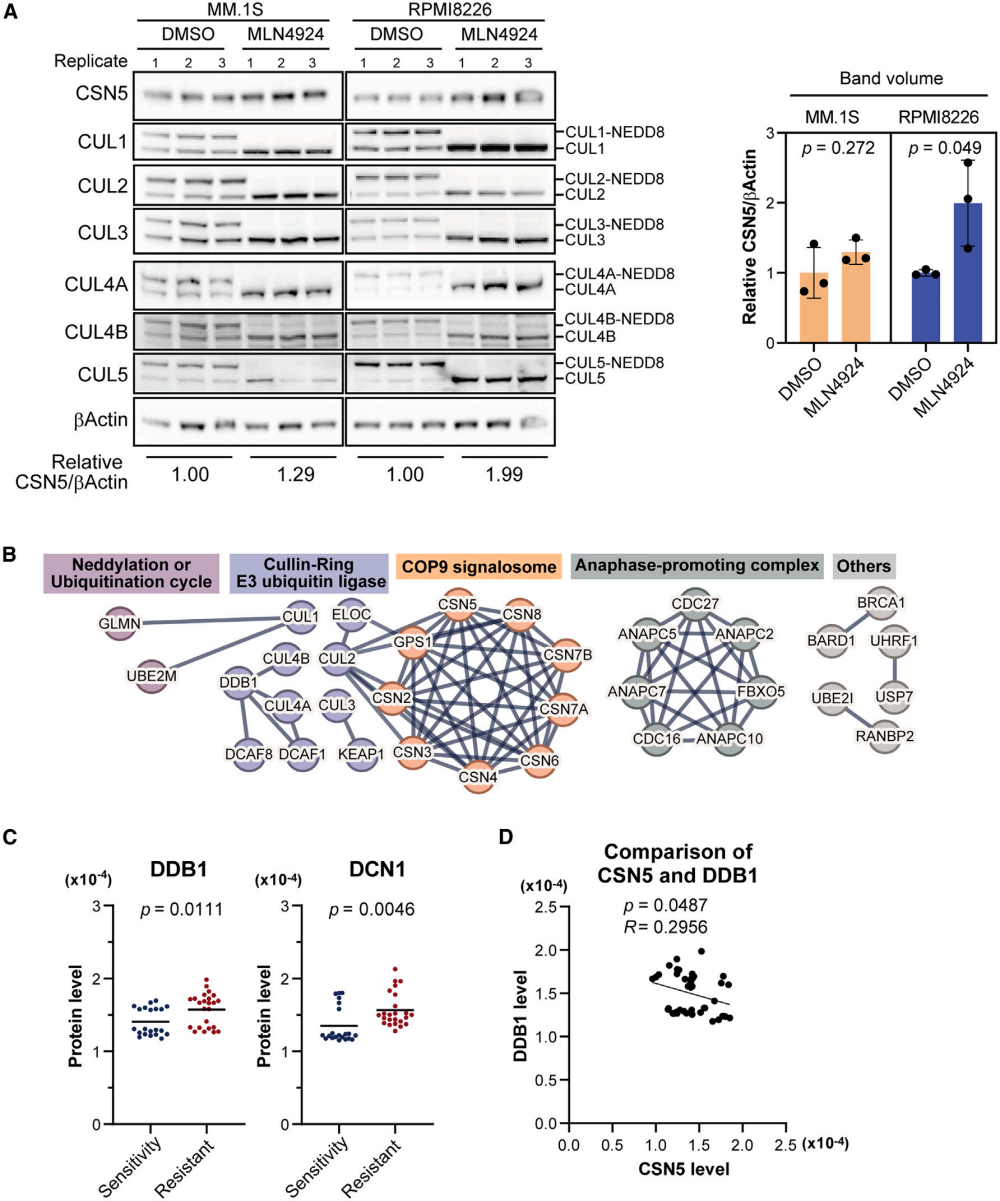
E3 ubiquitin ligase is responsible for CSN5 ubiquitination (A) Western blots representing CSN5, CUL1, CUL2, CUL3, CUL4A, CUL4B, and CUL5 expression levels in MM.1S and RPMI8226 treated with DMSO or 10 μM MLN4924 for 12 h. This experiment was performed in triplicate. β-Actin was used as a loading control. Band densities were quantified using iBright Analysis Software. Data are represented as mean ± standard deviation. Student's t test. (B) Proximity proteomics was performed using RPMI8226 cells overexpressing APEX2-CSN5 or TurboID-CSN5. We identified proteins that were highly enriched (2-fold or more, *p* < 0.01) in biotin-tyramide- or biotin-treated samples as proximal proteins of CSN5. We selected ubiquitin-related proteins from the enriched proteins and performed an STRING analysis (https://string-db.org/). A protein interaction network generated using STRING is shown. (C) Comparison of the levels of DDB1 and DCN1 expression quantified in proteomics analysis of LEN-sensitive and resistant cell groups among the 15 MM cell lines. Student's t test. (D) Comparison of the protein levels between CSN5 and DDB1 quantified in proteomics analysis of 15 MM cell lines.

We next attempted to identify the proteins that ubiquitinate CSN5 in RPMI8226 cells. We hypothesized that CSN5 is ubiquitinated by CRLs because CRLs are the largest family of E3 ubiquitin ligases.^11^ Furthermore, previous studies using human embryonic kidney cells indicated the involvement of CUL4B or CUL5 in the ubiquitination of CSN5.^32^^,^^33^ To investigate this hypothesis, we performed a ubiquitination inhibition assay using MLN4924, which inhibits CRL activity by suppressing neddylation. Upon the addition of MLN4924, the levels of neddylated cullin 1, 2, 3, 4A, 4B, and 5 were dramatically decreased, whereas CSN5 levels were increased by 1.29-fold (*p* = 0.272) and 1.99-fold (*p* = 0.049) in MM.1S and RPMI8226 cells, respectively (Figure 5A). This suggested that CRLs are involved in the ubiquitination of CSN5 in MM cells.

To elucidate the ubiquitination mechanism of CSN5, we performed proximity proteomics using RPMI8226 cells expressing APEX2-or TurboID-conjugated CSN5 (Figure S5A). It was expected that candidate E3 ubiquitin ligase complex components would be comprehensively identified because of the distinct labeling mechanisms of APEX2 and TurboID. In both the APEX2 and TurboID systems, biotinylated proteins were enriched in biotin-tyramide- or biotin-treated samples, whereas fewer proteins were enriched in biotin-tyramide- or biotin-free samples (Figure S5B). In proximity proteomics using APEX2 and TurboID, 1245 out of 3562 proteins and 1432 out of 5936 proteins were significantly enriched (*p* < 0.01) by more than 2-fold in the samples treated with biotin-tyramide or biotin, respectively (Figure S5C). Of the 3453 proteins identified in both groups, 3349 proteins were highly enriched in samples treated with biotin-tyramide and biotin (Figure S5D). Among the enriched proteins in the proximity proteomics using APEX2 or TurboID, 97 ubiquitin-related proteins were identified (Figure S5E). Based on a network analysis of the 97 proteins using STRING, interaction networks associated with CRLs, the COP9 signalosome, anaphase-promoting complex, neddylation or ubiquitination cycle, and other proteins were identified (Figure 5B). CUL1, CUL2, CUL3, CUL4A, and CUL4B were identified as cullin proteins, and we also identified adaptor and receptor proteins of CUL4A and CUL4B.

Next, we examined the trigger for CSN5 ubiquitination in RPMI8226 cells. It has been reported that the phosphorylation of CSN5 by phosphorylated IKKα or IKKβ activated by inflammatory signals induces the ubiquitination and degradation of CSN5.^33^ We compared the phosphorylation status of CSN5 between MM.1S and RPMI8226 cells by Phos-tag western blot analysis, but no difference in the band pattern was observed (Figure S6A). In addition, phosphorylated and total IKKα and IKKβ levels were not increased in RPMI8226 cells (Figure S6A). Therefore, CSN5 ubiquitination was not triggered by phosphorylated IKKα or IKKβ in RPMI8226 cells. Another possible reason for the enhanced CSN5 ubiquitination in LEN-resistant cells was increased expression of CRL components. However, the proteomic data showed no significant increases in the expression of CULs in the LEN-resistant cell lines compared to the sensitive cell lines (Figure S6B). In contrast, DDB1, an adaptor protein in CUL4A and CUL4B, was significantly upregulated in the resistant cell lines (Figure 5C) and was identified by proximity proteomics (Figure 5B). Interestingly, we found a significant negative correlation between DDB1 and CSN5 levels in the 15 cell lines (Figure 5D). The expression level of DCN1, which promotes neddylation, was also significantly increased in LEN-resistant cells (Figure 5C).

## Discussion

Our findings in this study revealed that in both LEN-resistant and LEN-sensitive MM cell lines, LEN sensitivity is induced in response to an overexpression of CSN5. Moreover, we established that overexpressed CSN5 enhances the activity of the CRBN axis, which is otherwise less in RPMI8226 cells. CSN5 is involved in several biological functions,^34^ such as the regulation of transcription factor specificity^35^ and cellular localization of proteins,^36^ as well as deneddylation. Here, we showed that the deneddylation activity of CSN5 is required for the enhancement of LEN sensitivity. The incorporation of CSN5 into the COP9 signalosome is essential for deneddylation activity.^29^ Interestingly, deneddylation activity was increased by overexpression of only CSN5, although other COP9 signalosome proteins were not increased by CSN5 overexpression. These findings suggest that CSN5 is not involved in the regulation of expression of other COP9 signalosome proteins and CSN5 expression is the rate-limiting factor for deneddylation activity in MM cells. We believe that targeting CSN5 is sufficient for enhancement of the intracellular deneddylation activity.

There are several reports on the relationship between the COP9 signalosome and LEN resistance. Sievers et al. and Liu et al. reported that the artificial downregulation of COP9 signalosome proteins conferred resistance to LEN in MM cell lines.^20^^,^^21^ By combining proteomics and LEN sensitivity assay data from 15 MM cell lines, we found a decrease in the endogenous CSN5 levels in LEN-resistant MM cell lines. Moreover, we detected a negative correlation between CSN5 levels and cell viability in the presence of LEN. These data suggest that CSN5 expression is a promising biomarker for LEN sensitivity. In addition, the identification of RRM1^18^ and EDC4,^21^ which are reportedly associated with LEN resistance, by the combination analysis suggests that the proteomics data were useful for identifying proteins involved in LEN resistance. In this study, we used 15 MM cell lines without LEN treatment to identify the mechanisms of primary resistance. The expression of CRBN did not correlate with LEN sensitivity, consistent with a previous report.^37^ This result indicates that there is no correlation between CRBN expression and primary LEN sensitivity.

**Figure 6:**
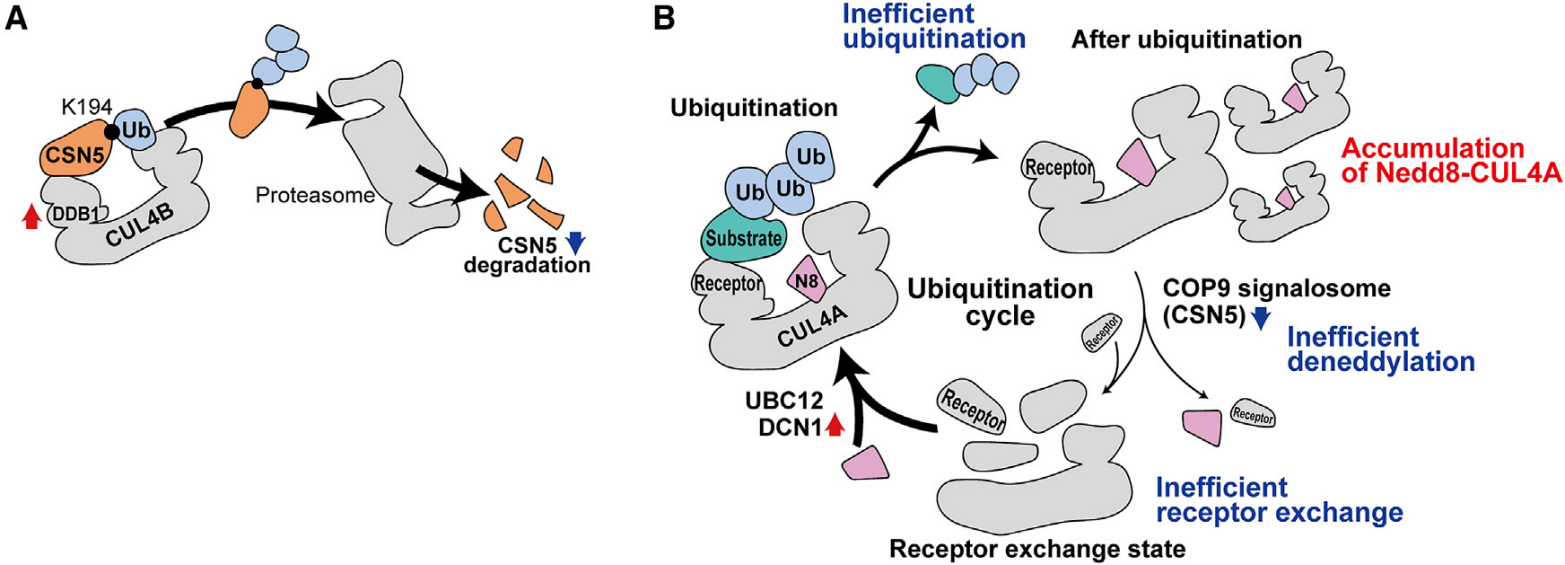
Hypotheses of CSN5 reduction in LEN-resistant cell lines and mechanisms by which low CSN5 expression of induces LEN resistance (A) Hypothesis of CSN5 degradation in LEN-resistant MM cells. K194 of CSN5 is ubiquitinated by CRL4B containing DDB1 and degraded by the proteasome. Increased expression of DDB1 in LEN-resistant cells is presumed to promote the ubiquitination of CSN5 and reduce the CSN5 level. (B) Hypothesis of the LEN resistance mechanism in LEN-resistant cells with low CSN5 expression. The low level of CSN5 in LEN-resistant cells induces an abnormal ubiquitination cycle of CRL4A, which inhibits receptor exchanges in the CRL complex. Consequently, the incorporation efficiency of CRBN into CRL4A is decreased, resulting in inhibition of the ubiquitination of IKZF1 and IKZF3 upon LEN treatment.

Our study demonstrated that K194 of CSN5 is highly ubiquitinated in two LEN-resistant cell lines, leading to the degradation of CSN5. K194R mutation did not completely stop the degradation, which indicates that other ubiquitination sites are also required for degradation. Ubiquitination of a protein at different sites involves E3 ligase specificity and protein modification.^38^^,^^39^^,^^40^ Multiple ubiquitination sites on a protein are involved in its degradation.^41^ The difference in CSN5 ubiquitination might account for the difference in degradation of this protein in the two cell lines. In this study, CRL is suggested to be involved in the CSN5 degradation process. However, it remains unclear what triggers CSN5 degradation and why a low CSN5 level results in LEN resistance. CSN5 is reported to be ubiquitinated by CRL4B,^32^ and we identified CUL4B and its adaptor protein DDB1 as proximal proteins of CSN5. In the CUL4-DDB1 E3 ubiquitin ligase complex, substrate ubiquitination is regulated by the interaction between DDB1 and the receptor protein.^42^ The degradation activity of CUL4-DDB1 E3 ubiquitin ligases is also regulated by the expression level of DDB1.^42^ In this study, DDB1 expression was higher in the LEN-resistant cell lines than in the sensitive cell lines. In addition, a negative correlation was observed between DDB1 and CSN5 levels in the 15 cell lines. These data suggest that CSN5 is ubiquitinated by the CUL4B-DDB1 E3 ubiquitin ligase complex and that the ubiquitination level depends on the DDB1 level in MM cell lines (Figure 6A).

To the best of our knowledge, there have been no previous studies reporting the mechanisms whereby a reduction in the expression of CSN5 promotes the induction of LEN resistance. As COP9 signalosome inactivates CUL4A via deneddylation, it can be speculated that a reduction in the COP9 signalosome contributes to an enhancement of LEN sensitivity. However, converse findings have also been reported, indicating that a reduction in the components of the COP9 signalosome is associated with LEN resistance. Moreover, our findings in the present study provide evidence to indicate that an increase in COP9 signalosome activity enhances LEN sensitivity. At present, however, the reasons for these discrepant findings remain unclear. Liu et al. reported a mechanism by which reduced CSN6 expression induces LEN resistance.^20^ They suggested that the downregulation of CSN6 induces a decrease in deneddylation activity, leading to an increase in neddylated CUL1, which in turn promotes the degradation of CRBN through ubiquitination, contributing to LEN resistance. However, we found no correlation between CRBN levels and LEN sensitivity in the 15 MM cell lines, suggesting that LEN sensitivity is independent of the CRBN level in these cell lines. As neddylation and deneddylation are required for the CRL ubiquitination cycle^43^ and for CRL complex reassembly in plants,^44^ we hypothesize that an aberration in the ubiquitination cycle of CRL4A is one of the reasons for LEN resistance mediated by low CSN5 (Figure 6B). In the ubiquitination cycle, CRL is deneddylated by the COP9 signalosome after ubiquitination of the substrate, leading to the dissociation of the CRL complex.^43^ Subsequently, the CRL complex is reassembled by neddylation by UBC12, after which CRL ubiquitinates a new substrate. This ubiquitination cycle promotes exchanges of the adaptor and substrate receptors, allowing the ubiquitination of a wide range of substrates.^43^ Neddylation by UBC12 has been shown to be enhanced through its interaction with DCN1.^45^^,^^46^^,^^47^^,^^48^ In this study, the expression levels of CSN5 and DCN1 were lower and higher in the LEN-resistant cell lines, respectively, than in the LEN-sensitive cell lines. These findings suggest that an increase in the assembly of CRL4A along with a reduced disassembly efficiency results in the accumulation of neddylated CUL4A and a decreased efficiency of CRBN incorporation into CRL4A. In agreement with this hypothesis, neddylated CUL4A was accumulated in RPMI8226 cells compared to MM.1S cells. Although further studies are required, these results support our hypothesis that an aberrant ubiquitination cycle reduces the ubiquitination activity of CRL4A, thereby inducing LEN resistance.

In conclusion, we demonstrated that LEN sensitivity can be induced by upregulation of CSN5. We proposed a hypothesis to account for the current inconsistent findings regarding the association between levels of the COP9 signalosome and LEN sensitivity. Combining LEN with drugs that selectively inhibit CSN5 ubiquitination or enhance the deubiquitination of K194 on CSN5 may be effective therapeutic strategies to overcome LEN resistance.

### Limitations of the study

Although we showed that LEN sensitivity can be induced by upregulation of CSN5, it was considered that CSN5 restoration could not overcome the resistance induced by the mutations in the CRBN axis-related genes because these mutations deregulate the CRBN-IKZF1/3-IRF4 axis downstream of CSN5 regulation. Therefore, to induce CSN5 expression and enhance LEN sensitivity, it is necessary to first investigate mutations in these genes.

Additional analyses are required to determine whether the phenomena revealed in this study occur in clinical specimens. However, because multiple drugs, including LEN, are commonly administered to treat MM, it is difficult to obtain tumor cells that are resistant specifically to LEN.

CSN5 is known as an oncogene, and high CSN5 expression has been reported to induce malignant transformation in some cancers.^49^^,^^50^ Although there are no reports of high CSN5 expression affecting cancer malignancy in MM to date, CSN5 levels will need to be strictly controlled to ensure that they do not become too high for safety reasons.

## Resource availability

### Lead contact

Further information and requests should be directed to the lead contact, Takeshi Masuda (t-masuda.z3@keio.jp).

### Materials availability

This study did not generate new unique reagents.

### Data and code availability

• Raw proteomic data files and original data of western blots are deposited in jPOST and Mendelay, respectively. This information is listed in the key resources table.
• This paper does not report the original code.
• All other data reported in this paper will be shared by the lead contact upon request.

## Acknowledgments

We thank all members of our laboratory for help and discussions. We also thank Koshi Imami for kindly providing the plasmid. This work was funded by 10.13039/501100001691JSPS KAKENHI (grant nos. 19K05544, 22H05546, and 22H02604).

## Author contributions

T.Y., S.I., S.O., and T.M. supervised the study. T.Y., A.F., H.O., K.Y., and T.M. prepared the samples. Z.Y. collected the western blot data of Phos-tag. T.Y., A.F., S.K., and T.M. performed the biochemical analysis. T.Y., A.F., N.A., and T.M. performed the proteomics analyses. N.K. constructed the website for the MM proteome data. All authors contributed to manuscript and figure preparation.

## Declaration of interests

The authors declare no competing interests.

## STAR★Methods

### Key resources table

**Table:** 

REAGENT or RESOURCE	SOURCE	IDENTIFIER
**Antibodies**		

Anti-Ikaros (IKZF1) Antibody	Cell Signaling Technology	Cat#5443; RRID: AB_10691693
Anti-Aiolos (IKZF3) Antibody, clone 9D10	Millipore	Cat#MABE911
Anti-rabbit IgG, HRP-linked Antibody	Cell Signaling Technology	Cat#7074; RRID: AB_2099233
Anti-β-Actin pAb-HRP-DirecT	MBL Life Science	Cat#PM053-7; RRID: AB_10697035
CSN5 Rabbit pAb	Cell Signaling Technology	Cat#6895; RRID: AB_10839271
CUL1 Rabbit pAb	Cell Signaling Technology	Cat#4995; RRID: AB_2261133
CUL2 (C-4) Mouse mAb	Santa Cruz Biotechnology	Cat#sc-166506; RRID: AB_2230072
CUL3 Rabbit pAb	Cell Signaling Technology	Cat#2759; RRID: AB_2086432
CUL4A Rabbit pAb	abcam	Cat#ab72548; RRID: AB_1268363
CUL4B Rabbit pAb	Proteintech	Cat#12916-1-AP; RRID: AB_2086699
CUL5 (F-6) Mouse mAb	Santa Cruz Biotechnology	Cat#sc-373822; RRID: AB_10992228
FLAG Mouse mAb, M2	SIGMA	Cat#F3165; RRID: AB_259529
Goat anti-Mouse IgG (H + L) Secondary Antibody, HRP	Thermo Fisher Scientific	Cat#62–6520; RRID: AB_88369
IKKα Rabbit pAb (H-744)	Santa Cruz Biotechnology	Cat#sc-7218; RRID: AB_2079399
IKKβ Rabbit pAb	Cell Signaling Technology	Cat#2684; RRID: AB_2122298
IRF4 Rabbit pAb	Cell Signaling Technology	Cat#4964; RRID: AB_10698467
LC3 Mouse mAb	MBL Life Science	Cat#M152-3; RRID: AB_1279144
Phospho-IKKα (Ser176)/IKKβ (Ser177) (C84E11) Rabbit mAb	Cell Signaling Technology	Cat#2078; RRID: AB_2079379
Phospho-IKKα/β (Ser176/180) (16A6) Rabbit mAb	Cell Signaling Technology	Cat#2697; RRID: AB_2079382
Ubiquitin (P4D1) Mouse mAb	Santa Cruz Biotechnology	Cat#sc-8017; RRID: AB_628423

**Chemicals, peptides, and recombinant proteins**		

2-chloroacetamide	Wako	Cat#032-09762
Dithiothreitol	Wako	Cat#049-08972
MG132	Selleck	Cat#S2619
MLN4924	Selleck	Cat#S7109
Lenalidomide	Selleck	Cat#S1029
Bafilomycin A1	Selleck	Cat#S1413
Cycloheximide	Wako	Cat#035-20992
Lysyl Endopeptidase	Wako	Cat#121-05063
Sequencing Grade Modified Trypsin	Promega	Cat#V5113
Sodium deoxycholate	Wako	Cat#194-08311
Sodium lauroyl sarcosinate	Wako	Cat#196-10385

**Critical commercial assays**		

BCA Protein Assay Kit	Thermo Fisher Scientific	Cat #23227
PTMSscan HS K-e-GG Remnant Magnetic Immunoaffinity Beads	Cell Signaling Technology	Cat#5562

**Deposited data**		

Mass spectrometry data	This paper	jPOST/ProteomeXchange: JPST002950/PXD050020, JPST003373/PXD056174
Original western blot images	This paper	Mendeley: https://doi.org/10.17632/ggpw7kjjdb.1
Proteomics data of 15 MM cell lines	This paper	MM Proteome Data: https://mmproteomicsdata.iab.keio.ac.jp/

**Experimental models: Cell lines**		

KMS11	JCRB Cell Bank	Cat#JCRB1642
KMS-12BM	JCRB Cell Bank	Cat#JCRB0429
Lenti-X 293T	Takara bio	Cat#632180
MM.1S	ATCC	Cat#CRL-2974
RPMI8226 (Wild type)	ATCC	Cat#CCL-155
RPMI8226, CSN5-H138Q	This study	N/A
RPMI8226, CSN5-K194R	This study	N/A
RPMI8226, Vehicle	This study	N/A
RPMI8226, Wild type-CSN5	This study	N/A

**Oligonucleotides**		

CSN5-qPCR-F	PrimerBank ID: 38027922c1	TGGGTCTGATGCTAGGAAAGG
CSN5-qPCR-R	PrimerBank ID: 38027922c1	CTATGATACCACCCGATTGCATT
GAPDH-qPCR-F	Cheng et al.[1]	GGAGCGAGATCCCTCCAAAAT
GAPDH-qPCR-R	Cheng et al.[1]	GGCTGTTGTCATACTTCTCATGG

**Recombinant DNA**		

lentiCas9-Blast	Addgene	Cat#52962
lentiEF1-FLAG-APEX2-CSN5-P2A-Blast	This study	N/A
lentiEF1-FLAG-CSN5-P2A-Blast	This study	N/A
lentiEF1-FLAG-P2A-Blast	This study	N/A
lentiEF1-FLAG-TurboID-CSN5-P2A-Blast	This study	N/A
pEXP-MFGE8-APEX2	Dr Koshi Imami	N/A
pMD2.G	Addgene	Cat#12259
psPAX2	Addgene	Cat#12260
TurboID-His6_pET21a	Addgene	Cat#107177

**Software and algorithms**		

DIA-NN 1.8.1	Dr Markus Ralser	https://github.com/vdemichev/DiaNN
GraphPad Prism 8	GraphPad Software Inc.	http://www.graphpad.com/
iBright Analysis Software	Thermo Fischer Scientific	https://www.thermofisher.com/
Illustrator	Adobe	http://www.adobe.com/
Metascape	Website	https://metascape.org/
Msconvert version 3.0.22248	ProteomeWizard	https://proteowizard.sourceforge.io/
Photoshop	Adobe	http://www.adobe.com/
Proteome Discioverer 2.5	Thermo Fischer Scientific	https://www.thermofisher.com/
SIMCA 17.0	SARTORIUS	https://www.sartorius.com/
STRING	Website	https://string-db.org/

### Experimental model and study participant details

#### Materials

Roswell Park Memorial Institute (RPMI) 1640 medium, Dulbecco’s modified Eagle’s medium (DMEM), sample buffer solution (2ME-), Tris, sodium deoxycholate (SDC), sodium lauroyl sarcosinate (SLS), Phos-tag sodium dodecyl sulfate (SDS) polyacrylamide gel, dithiothreitol (DTT), iodoacetamide (IAA), 2-chloroacetamide (CAA), lysyl endopeptidase (Lys-C), biotin, blasticidin, and cycloheximide were obtained from Fujifilm Wako (Osaka, Japan). In-Fusion HD Cloning Kit, Lenti-X Concentrator, Western BLoT Hyper HRP Substrate, and 2.5 mM dNTPs were obtained from TaKaRa Bio (Kusatsu, Japan). THUNDERBIRD SYBR qPCR Mix, KOD -Plus- Mutagenesis Kit, and Can Get Signal Solution were purchased from TOYOBO (Osaka, Japan). LDS sample buffer, BCA Assay Kit, 660nm Protein Assay Kit, Oligo(dT)20 Primer, RNaseOUT Recombinant Ribonuclease Inhibitor, SuperScript III Reverse Transcriptase, and streptavidin magnetic beads were purchased from Thermo Fisher Scientific (San Jose, CA, USA). MLN4924, LEN, MG132, PR619, and bafilomycin A1 were obtained from Selleck (Houston, TX, USA). 1 M triethylammonium bicarbonate buffer (TEAB; pH 8.5), phosphatase inhibitor cocktail, and protease inhibitor cocktail were purchased from Sigma-Aldrich (St. Louis, MO, USA).

#### Cell culture

RPMI8226, MM.1S, U266, and H929 cells were obtained from the American Type Culture Collection (Manassas, VA, USA). KMS11, KMS12BM, KMS12PE, KMS20, KMS27, KMS28BM, KMS28PE, and KMM1 cells were obtained from Japanese Collection of Research Bioresources Cell Bank (Osaka, Japan). L363 and MOLP8 cells were obtained from the German Collection of Microorganisms and Cell Cultures (DSMZ, Braunschweig-Süd, Germany). OPM1 cells were gifted by Edward Thompson (University of Texas, Galveston, TX). Lenti-X 293T cells were purchased from TaKaRa Bio. Human MM cells were cultured in RPMI 1640 medium supplemented with 10% fetal calf serum (FCS; Biowest, Nuaillé, France), 100 U/mL penicillin, and 100 μg/mL streptomycin at 37°C in the presence of 5% CO_2_. Lenti-X 293T cells were maintained in DMEM supplemented with 10% (v/v) FCS, 100 U/mL penicillin, and 100 μg/mL streptomycin at 37°C in the presence of 5% CO_2_.

### Method details

#### Vector construction and mutagenesis

Full-length human *CSN5* cDNA was synthesized from mRNA isolated from RPMI8226 cells. mRNA was extracted using an RNeasy kit (Qiagen, Hilden, Germany) and cDNA was synthesized by reverse transcription using Oligo(dT)20 Primer, RNaseOUT Recombinant Ribonuclease Inhibitor, 2.5 mM dNTPs, and SuperScript III Reverse Transcriptase. The *CSN5* cDNA was cloned into lentiEF1-FLAG-P2A-Blast using an In-Fusion HD Cloning Kit. The lentiEF1-FLAG-P2A-Blast vector was used as an Empty vector. lentiEF1-FLAG-P2A-Blast was constructed by removing the Cas9 sequence from lentiCas9-Blast (Addgene plasmid #52962; http://n2t.net/addgene:52962; RRID:Addgene_52962) using an In-Fusion HD Cloning Kit. A KOD -Plus- Mutagenesis Kit was used to replace the 138th histidine of CSN5 with a glutamine and the 194th lysine with an arginine. For proximity proteomics, the TurboID and APEX2 genes were subcloned from TurboID-His6_pET21a (Addgene plasmid # 107177; http://n2t.net/addgene:107177; RRID:Addgene_107177) and pcDNA5/FRT/MFGE8-APEX2 (kind gift from Dr. Koshi Imami) into lentiEF1-FLAG-CSN5-P2A-Blast using an In-Fusion HD Cloning Kit.

#### Lentiviral transduction

HEK293T cells were cultured in DMEM supplemented with 10% FCS, 100 U/mL penicillin, and 100 μg/mL streptomycin at 37°C in the presence of 5% CO_2_. When the cells reached 80% confluence in a 100-mm dish, they were transfected with 10 μg of lentiviral plasmid, 3.5 μg of psPAX2 (Addgene plasmid # 12260; http://n2t.net/addgene:12260; RRID:Addgene_12260), and 6.5 μg of pMD2.G (Addgene plasmid # 12259; http://n2t.net/addgene:12259; RRID:Addgene_12259) using PEIpro (Polyplus-transfection, Ilkirch, France). The lentivirus-containing supernatant was harvested at 48 h and 72 h after transfection and centrifuged at 500 × *g* at 37°C for 10 min. The supernatant was filtered through a 0.45-μm membrane filter. To concentrate the lentiviruses, the filtrate containing the lentiviruses was incubated with Lenti-X Concentrator at 4°C for 1 h and then centrifuged at 1500 × *g* at 4°C for 45 min. After removal of the supernatant, the lentiviral pellet was reconstituted with DMEM and used for spin infection. For spin infection, MM cells were seeded in a 12-well plate (at 1 × 10^6^ cells/well) in RPMI1640 medium supplemented with 7 μg/mL polybrene, and the reconstituted viruses were added to the cells. The plate was centrifuged at 900 × *g* at 37°C for 2 h. Then, 1 mL of supernatant was removed and 1 mL of fresh RPMI1640 medium was added.

#### Western blot analysis

Denatured proteins were separated on 8% or 10% Bolt Bis-Tris Plus Mini Protein Gels in MOPS or MES Running Buffer and transferred to polyvinylidene difluoride membranes using a Power Blotter System or iBlot2 (all from Thermo Fischer Scientific). The membranes were blocked with 5% skim milk in TBS-T for 60 min and probed with primary antibodies. All primary antibodies were diluted 500–5000 times with Can Get Signal Solution 1. The membranes were washed with TBS-T and then incubated with horseradish peroxidase (HRP)-conjugated anti-mouse IgG or anti-rabbit IgG in Can Get Signal Solution 2. The immunocomplexes were visualized using Western BLoT Hyper HRP Substrate. The signals were detected using an Omega Lum G imaging system (Gel Company, San Francisco, CA) or iBright CL1500 Imaging System (Thermo Fischer Scientific). Band densities were quantified using iBright Analysis Software (Thermo Fischer Scientific).

#### LEN sensitivity assay

LEN was added to 5 × 10^4^ cells at a final concentration of 0.01, 0.1, 1, 10, or 100 μM in 25-cm^2^ cell-culture flasks. The cells were incubated at 37°C for 5 or 6 days, and the number of viable cells was determined using a Cell Counting Kit-8 (Dojindo, Kumamoto, Japan).

#### CHX assay

CHX was added to 1.0 × 10^6^ cells at a final concentration of 10 μM in a 25-cm^2^ cell-culture flask and incubated at 37°C in the presence of 5% CO_2_. The cell pellets were dissolved in LDS sample buffer and subjected to sonication in an ice bath for 30 min. Then, the samples were incubated at 70°C for 10 min and subjected to western blot analysis.

#### CSN5 accumulation assay after CUL inhibition with MLN4924

MLN4924 was added to 1 × 10^6^ cells at a final concentration of 10 μM in a 25-cm^2^ cell-culture flask. The cells were incubated at 37°C for 12 h. Cell pellets were dissolved in LDS sample buffer and subjected to sonication in an ice bath for 30 min. Then, the samples were incubated at 70°C for 10 min and subjected to western blot analysis.

#### Gene expression analysis using qPCR

mRNA extraction and cDNA synthesis were performed as mentioned above. qPCRs were run in a QuantStudio 3 system (Thermo Fisher Scientific) using THUNDERBIRD SYBR qPCR Mix. Transcript levels were calculated relative to the *GAPDH* level in each sample. The qPCR primer sequences were: *CSN5* forward: 5′-TGGGTCTGATGCTAGGAAAGG-3′, *CSN5* reverse: 5′-CTATGATACCACCCGATTGCATT-3′, *GAPDH* forward: 5′-GGAGCGAGATCCCTCCAAAAT-3′, *GAPDH* reverse: 5′-GGCTGTTGTCATACTTCTCATGG-3′.

#### Evaluation of the CSN5 degradation pathway

MG132 or bafilomycin A1 was added to 1.0 × 10^6^ cells at a final concentration of 5 μM or 100 μM in 75-cm^2^ cell-culture flasks. The cells were incubated at 37°C in the presence of 5% CO_2_ for 8 h. Cellular proteins were extracted using LDS sample buffer and sonication in an ice bath for 30 min. The protein samples were incubated at 70°C for 10 min and subjected to western blot analysis.

#### Comparison of CSN5 phosphorylation status in MM.1S and RPMI8226 cells

MM.1S and RPMI8226 cells were cultured to 80% confluence. Proteins were extracted using LDS sample buffer containing phosphatase inhibitor cocktail. The acrylamide pendant Phos tag ligand and two equivalents of ZnCl_2_ were added to the separating gel before polymerization. The running buffer consisted of 100 mM Tris and 100 mM MOPS containing 0.1% SDS and 5 mM sodium bisulfite. After electrophoresis, the gel was washed twice with a solution containing 25 mM Tris, 192 mM glycine, 10% methanol, and 1.0 mm EDTA for 20 min and then washed once with a solution containing 25 mM Tris, 192 mM glycine, and 10% methanol for 20 min. Gel transfer, blocking, antibody reactions, and detection were performed according to the normal immunoblotting protocol.

#### Proteomics analysis of CSN5-overexpressing RPMI8226 cells

Cells were harvested and washed twice with cold PBS. Proteins were extracted using PTS buffer (12 mM SDC, 12 mM SLS, 100 mM TEAB) and quantified using the BCA Assay Kit. We used 10 μg proteins for digestion. Reduction and alkylation were performed by incubation with 10 mM DTT and 50 mM CAA, respectively. The proteins were digested with Lys-C followed by trypsin at 37°C for 14 h. The SDC and SLS were removed using a phase transfer method.^51^^,^^52^ Peptides were purified using GL-Tip SDB (GL Sciences, Tokyo Japan) and analyzed by liquid chromatography-tandem mass spectrometry (LC-MS/MS).

#### Ubiquitin proteomics analysis

MG132 was added to 1.0 × 10^7^ MM.1S, RPMI8226, KMS11, or KMS27 cells at a final concentration of 5 μM and the cells were incubated for 8 h. Cell pellets were suspended in a cell lysis buffer for ubiquitin proteomics (5% SDS, 50 mM TEAB) and the proteins were quantified using the BCA Assay Kit. The protein concentrations were adjusted to 1 mg/500 μL using the cell lysis buffer for ubiquitin proteomics. The proteins were reduced and alkylated with 5 mM DTT and 10 mM IAA and then digested using an S-Trap Midi column (ProtiFi, Fairport, NY, USA). The samples were conditioned by the addition of 50 μL of 12% phosphoric acid and 3.3 mL of S-Trap buffer (90% methanol in 50 mM TEAB). The conditioned sample solutions were loaded into the S-Trap midi column. The column was washed three times with S-Trap buffer and then, 500 μL of digestion solution containing 10 μg of trypsin (Promega, Madison, MA, USA) and 50 mM TEAB was added to digest the proteins in the column at 37°C for 14 h. Peptides were recovered from the column by sequential washing with 50 mM TEAB, 0.5% TFA, and 50% acetonitrile containing 0.5% TFA. Peptides with the ubiquitin remnant motif (K-ε-GG) were pulled down using PTMScan HS K-ε-GG Remnant Magnetic Immunoaffinity Beads (Cell Signaling Technology) on a rotator at 4°C for 2 h. The beads were washed thrice with PTMScan HS IAP wash buffer and twice with cold water. The peptides were eluted with 0.5% TFA, purified using GL-Tip SDB, and analyzed by LC-MS/MS.

#### Proximity labeling with TurboID

Proximity labeling with TurboID was performed as previously reported.^53^ Briefly, cells were cultured in DMEM supplemented with 10% dialyzed FBS (Biowest) and 1% penicillin/streptomycin. Biotin was added to 2 × 10^7^ cells at a final concentration of 500 μM, and the cells were incubated at 37°C in the presence of 5% CO_2_ for 10 min. Proteins were extracted using RIPA lysis buffer (1 M Tris-HCl (pH 7.5), 150 mM NaCl, 10% SDS, 10% SDC, 10% Triton X-100) and concentrated using an Amicon Ultra-0.5 centrifugal filter with 3-kDa molecular weight cutoff (Millipore). Proteins were quantified using a BCA assay kit, and 1 mg of protein was added to streptavidin magnetic beads. The beads were washed once with 50 mM Tris-HCl (pH 7.5) and twice with 2 M urea in 50 mM Tris-HCl (pH 7.5), and then suspended in 270 μL of a suspension buffer (1 mM DTT, 2 M urea, 50 mM Tris-HCl (pH 7.5)). For western blotting, 27 μL of bead suspension was mixed with 80 μL of sample buffer (2ME-) containing 2 mM biotin and 20 mM DTT. Biotinylated proteins were extracted by shaking at 1000 rpm at 95°C for 10 min and subjected to western blot analysis.

#### Proximity labeling with APEX2

Proximity labeling with APEX2 was performed as previously reported.^54^^,^^55^^,^^56^ Cells were cultured in RPMI1640 medium supplemented with 10% FBS and 1% penicillin/streptomycin. Biotin-tyramide (BT, Iris Biotech GmbH, Marktredwitz, Germany) was added to 2 × 10^7^ cells at a final concentration of 500 μM, and the cells were incubated for 30 min. Then, H_2_O_2_ was added to cells at a final concentration of 1 mM. After incubation at room temperature for 1 min, the cells were washed three times with a quenching solution (10 mM sodium azide, 10 mM sodium ascorbate, 5 mM Trolox in PBS). Proteins were extracted using RIPA lysis buffer containing 1 mM sodium azide, 10 mM sodium ascorbate, and 1 mM Trolox. Proteins were quantified using a 660nm Protein Assay Kit, and 1 mg of protein was added to streptavidin magnetic beads. The beads were washed once with 1 mL of RIPA lysis buffer and twice with 2 M urea in 10 mM Tris-HCl (pH 8.0). Then, they were suspended in 270 μL of a suspension buffer (1 mM DTT, 2 M urea, 50 mM Tris-HCl (pH 7.5)). For western blotting, 27 μL of bead suspension was mixed with 80 μL of sample buffer (2ME-) containing 2 mM biotin and 20 mM DTT. Biotinylated proteins were extracted by shaking at 1000 rpm at 95°C for 10 min and subjected to western blot analysis.

#### Sample preparation for MS analysis of labeled proximal proteins

For protein digestion, 6 μg of trypsin was added to 243 μL of bead suspension, which was shaken at 1000 rpm at 25°C for 1 h. The supernatant was collected, and the beads were washed twice with 50 mM Tris-HCl (pH 7.5) containing 2 M urea to enhance the recovery of proteins and peptides. The sample solution was incubated with 4 mM DTT at 25°C for 30 min, followed by incubation with 1 mM 2-chloroacetamide at 25°C for 45 min. After reduction and alkylation, 6 μg of trypsin was added and the sample was incubated at 37°C for 15 h. After digestion, the peptides were acidified with trifluoroacetic acid and purified using GL-Tip SDB. The peptide solution eluted from GL-Tip SDB was dried using a centrifugal concentrator, and the peptides were resuspended in 75 μL of 0.1% TFA, 3% ACN, and 5 μL was subjected to LC-MS/MS.

#### NanoLC-MS/MS analysis

Proteomics of CSN5-overexpressing RPMI8226 cells was performed using an OrbiTrap Exploris mass spectrometer coupled with an Vanquish *Neo* UHPLC system (both from Thermo Fisher Scientific). Peptides were separated using a C18 packed emitter column (Nikkyo Technos, Tokyo, Japan). The flow rate was 300 nL/min. Data were acquired in the data-independent analysis (DIA) mode.

Ubiquitin proteomics was performed using an OrbiTrap Fusion Tribrid mass spectrometer coupled with an EASY-nLC 1200 system (both from Thermo Fisher Scientific). Peptides were loaded onto an Acclaim PepMap 100 C18 (Thermo Fisher Scientific) and separated on an C18 packed emitter column. The flow rate was 300 nL/min. Data were acquired in the data-dependent analysis (DDA) mode.

Proximity labeling proteomics based on APEX2 was performed using a TripleTOF 6600+ (Sciex, Framingham, MA, USA) coupled with a NanoLC400 (Eksigen). Proximity labeling proteomics based on TurboID was performed using a TripleTOF 7600 (Sciex) coupled with an Ultimate 3000 RSLC nano system (Thermo Fisher Scientific). Data were acquired in the DIA mode.

#### Data analysis

In the analysis of proteomic data obtained in triplicate for the 15 MM cell lines, MS raw data (JPST001390/PXD029814, JPST003373/PXD056174)^57^^,^^58^ deposited in the Japan Proteome Standard Repository database^59^ were downloaded. These 15 MM cells were cultured without lenalidomide,^57^^,^^58^ and these proteomics data were analyzed using Sequest HT in Proteome Discoverer 2.5 (Thermo Fisher Scientific) with the UniProt human reference database. Label-free quantification was performed using MS1 signal intensities of the identified peptides. For the analysis of 15 MM cell lines data, only proteins quantified in more than half of the total samples (i.e., 23) were used for comparative analysis. The MM cell lines were classified into LEN-sensitive and LEN-resistant groups based on the LEN sensitivity assay results. The proteomic data of the two groups were subjected to OPLS-DA and regression coefficients were calculated using SIMCA 17.0 (Sartorius Stedim Biotech, Umeå, Sweden). Protein expression levels obtained using Proteome Discoverer 2.5 were scaled to unit variance. The proteins with the top 10% and bottom 10% regression coefficient values were subjected to enrichment analysis using Metascape (https://metascape.org/gp/index.html#/main/step1).

In the analysis of proteomics data of CSN5-overexpressing RPMI8226 cells and proximity proteomics, DIA data were converted to the mzML format using MSconvert version 3.0.22248, and analyzed using DIA-NN 1.8.1^60^ with a library-free search. A spectral library was generated from the UniProt human reference database using DIA-NN. Protein levels were quantified using the MaxLFQ algorithm.^61^ For proximity proteomics, we identified proteins that were highly enriched (2-fold or more, *p* < 0.01) in biotin-tyramide- or biotin-treated samples as proximal proteins of CSN5. Proteins belonging to the “Ubiquitin System” (ko04121) in the KEGG BRITE Database (https://www.genome.jp/kegg/brite.html) were identified as ubiquitin-related proteins. For interaction network analysis, we used STRING (https://string-db.org/) with the following parameters: network type, physical type; meaning of network edges, confidence; active interaction sources, experiments; minimum required interaction score, highest confidence (0.900); network display options, hide disconnected nodes in the network.

In the analysis of ubiquitin proteomics data, proteins and ubiquitination sites were identified and quantified using MaxQuant 2.0.2.0 and the UniProt human reference database. Modification of lysine residues by GG was set as a variable modification. For protein quantification, we used the MaxLFQ algorithm.^61^

### Quantification and statistical analysis

The number of experimental repeats is indicated for each experiment or corresponding figure legend. For the proteomics data analysis, significant differences were assessed using Student’s t test in GraphPad Prism 8 (GraphPad Software, CA, USA) or Excel (Microsoft, WA, USA). Correlations between protein expression levels and cell viability were evaluated using simple linear regression in GraphPad Prism 8. Statistical significance was set at *p* < 0.05.

### Additional resources

The expression levels of the proteins identified by proteomics using the 15 MM cell lines and the correlations between the expression levels relative to LEN and cell viability are available at MM Proteome Data (https://mmproteomicsdata.iab.keio.ac.jp/).

## Supplemental information

Document S1. Figures S1–S6

Table S1. Proteome data of 15 MM cell lines, related to Figure 1
