# Supplementary material for: Increased CSN5 expression enhances the sensitivity to lenalidomide in multiple myeloma cells
Source: iScience. 2024 Nov 15;27(12):111399. doi: 10.1016/j.isci.2024.111399 (PMC11647120; doi:10.1016/j.isci.2024.111399)
Supplement: Supplementary file 1 — Document S1. Figures S1–S6 [file mmc1.pdf]

iScience, Volume *27*

Supplemental information

Increased CSN5 expression enhances the sensitivity to lenalidomide in multiple myeloma cells

Takumi Yamamoto, Arisu Furukawa, Yue Zhou, Nobuaki Kono, Shojiro Kitajima, Hiroto Ohguchi, Yawara Kawano, Shingo Ito, Norie Araki, Sumio Ohtsuki, and Takeshi Masuda

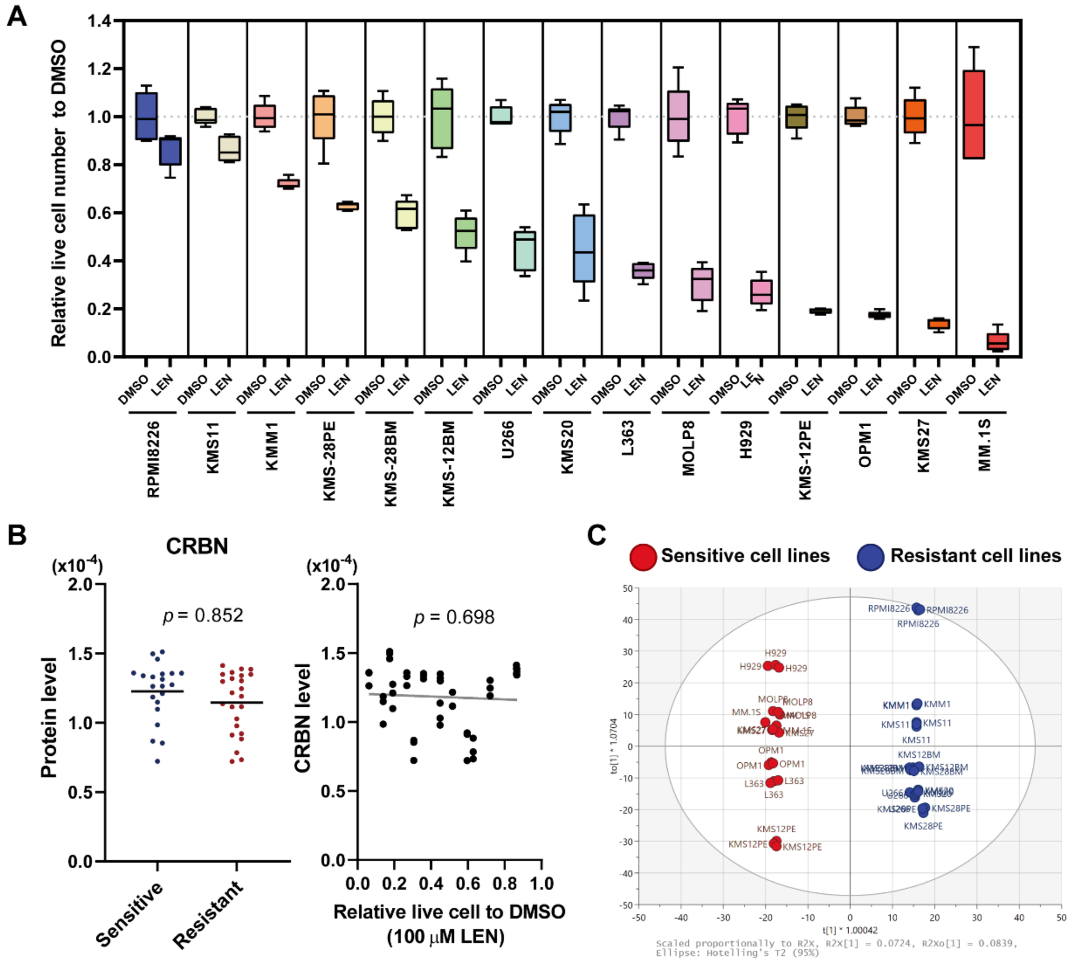

Figure S1 Lenalidomide (LEN) sensitivity and classification of 15 multiple myeloma cell lines, related to Figure 1

(A) Cell viability assay in LEN treated multiple myeloma cell lines. The cells were cultured for 6 days in the presence of 100 μM LEN, and viable cells were counted using a Cell Counting Kit. Error bar represents standard deviation.

(B) CRBN expression levels in LEN-sensitive and resistant cell lines (left panel). Relationship between LEN sensitivity and CSN5 expression levels in the 15 cell lines (right panel).

(C) Orthogonal partial least squares discriminant analysis (OPLS-DA) score plot. OPLS-DA analysis was performed using SIMCA v17.0.

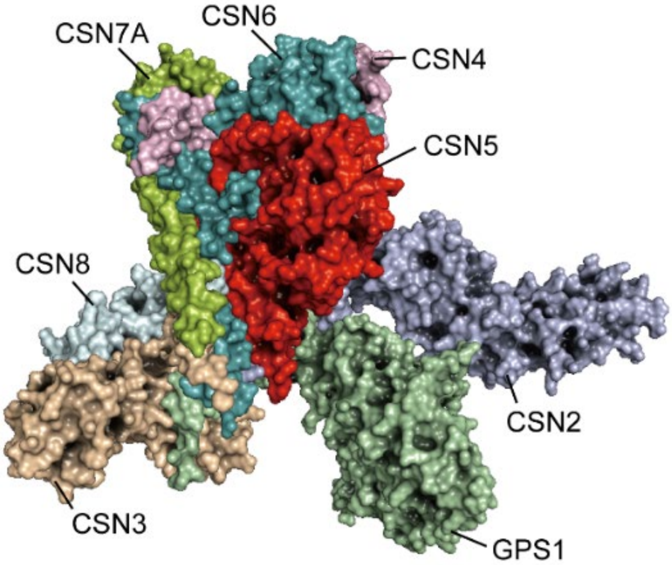

Figure S2 Structure of the COP9 signalosome complex, related to Figure 2

This structure was obtained from the Protein Data Bank (PDB: 4D10).

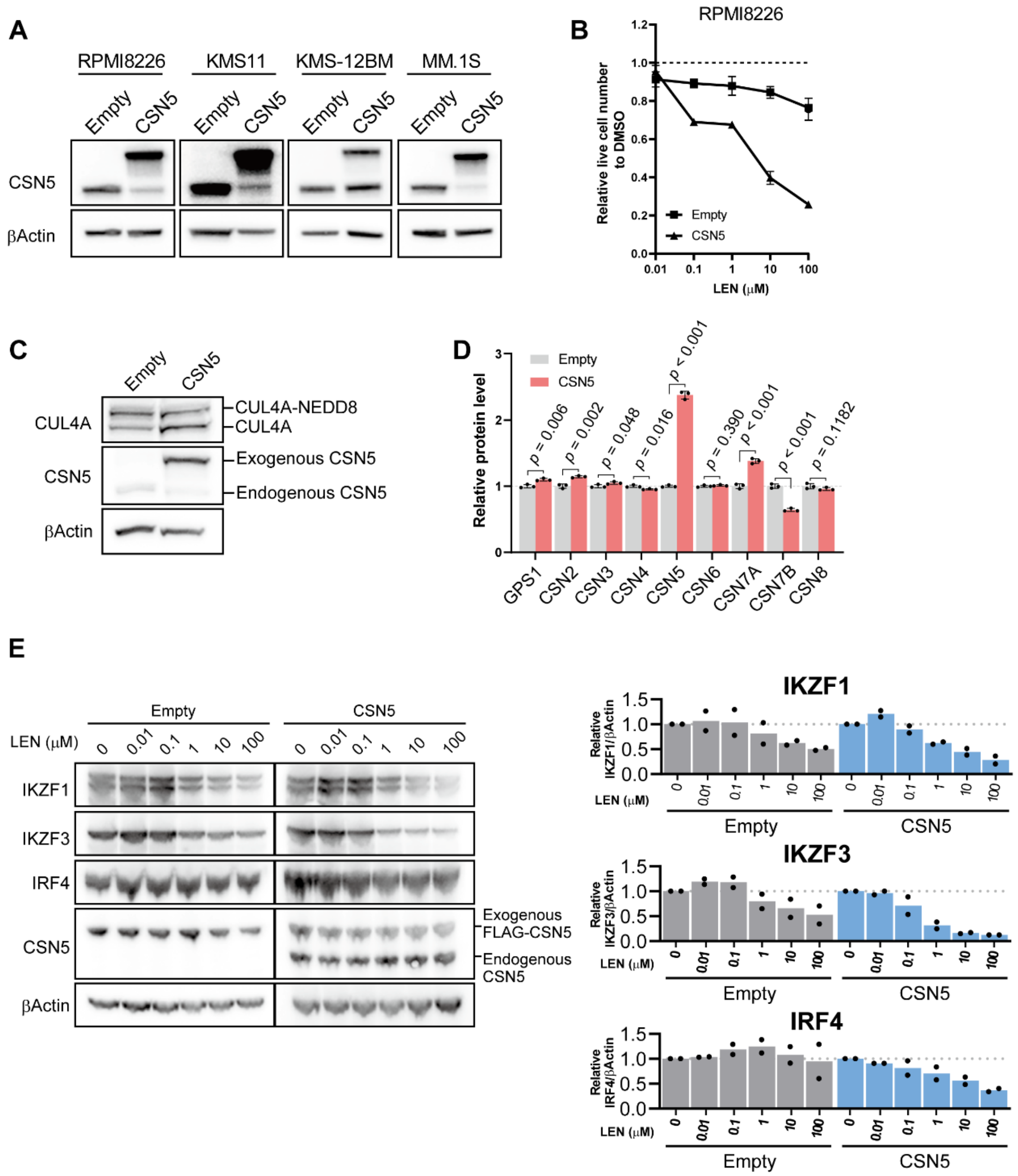

Figure S3 LEN sensitivity of multiple myeloma cell lines overexpressing CSN5, related to Figure 3

lentiEF1-FLAG-P2A-Blast was used as the Empty vector.

(A) Western blots representing the endogenous CSN5 and exogenous FLAG-CSN5 expression levels in RPMI8226, KMS11, KMS-12BM, and MM.1S cells. β-Actin was used as a loading control.

(B) Dependence of cell growth inhibition on LEN concentration in RPMI8226 cells overexpressing CSN5. The cells were cultured for 6 days in the presence of LEN, and viable cells

were counted using a Cell Counting Kit. Error bar represents standard deviation.

(C) Western blots representing the expression of CUL4A and CSN5 in RPMI8226 carrying empty vector and overexpressing FLAG-CSN5. β-Actin was used as a loading control. The smaller proportion of neddylated CUL4A suggested increased deneddylation activity in RPMI8226 cells overexpressing CSN5.

(D) Comparison of the expression levels of COP9 signalosome components between RPMI8226 cells carrying empty vector and RPMI8226 cells overexpressing FLAG-CSN5 by proteomics. The comparison was performed in triplicate. Data represent the mean ± standard deviation.

(E) Western blots of IKZF1, IKFZ3, IRF4, CSN5 expression levels after treatment with the indicated dose of LEN in FLAG-CSN5- or empty vector-overexpressing KMS-12BM cells. β-Actin was used as the loading control. The protein bands in western blotting of IKZF1, IKZF3 and IRF4 were quantified using iBright Analysis Software. Band densities of IKZF1, IKZF3 and IRF4 were normalized to that of β-actin. Each data represents the mean (n =2).

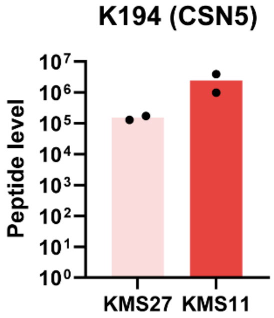

Figure S4 Comparison of ubiquitination level at K194 on CSN5 in KMS27 and KMS11 cells, related to Figure 4

Identification of ubiquitination sites on CSN5 were performed by proteomics. The experiment was conducted in technical duplicate.

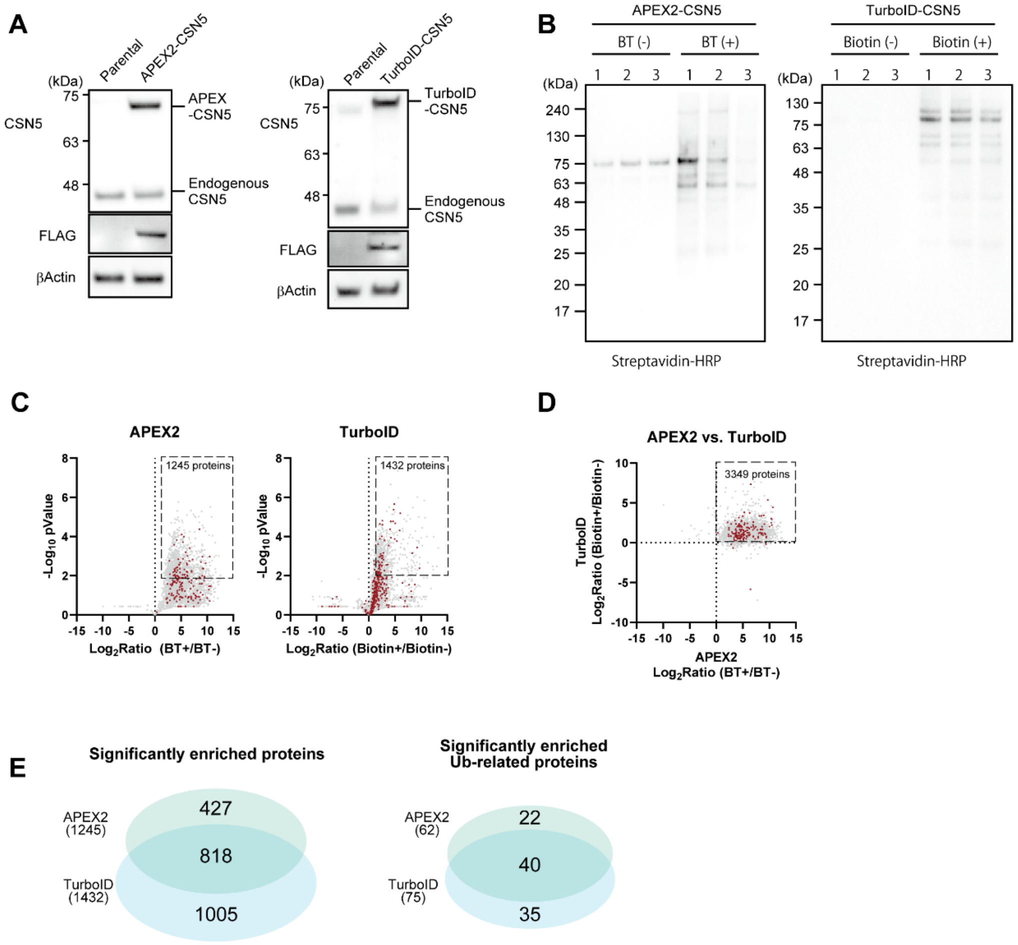

Figure S5 Identification of E3 ubiquitin ligase responsible for CSN5 ubiquitination, related to Figure 5

(A) Western blots representing endogenous CSN5, APEX2-CSN5 (left panel), or TurboID-CSN5 (right panel). β-Actin was used as a loading control.

(B) Western blots representing biotinylated proteins after enrichment by streptavidin beads using RPMI8226 cells expressing APEX2-CSN5 (left panel) or TurboID-CSN5 (right panel). These experiments were performed in triplicate. BT: biotin-tyramide.

(C) Volcano plots of proximity proteomics data in RPMI8226 cells expressing APEX2-CSN5 (left panel) or TurboID-CSN5 (right panel). Proximity proteomics was performed in triplicate. Red dots indicate ubiquitin-related proteins. BT: biotin-tyramide. We identified proteins significantly highly enriched (2-fold or more, $p < 0.01$) in biotin-tyramide- or biotin-treated samples as proximal proteins of CSN5.

(D) Comparison of the enrichment ratios of proteins commonly identified in proximity proteomics using RPMI8226 cells expressing APEX2-CSN5 and TurboID-CSN5. BT: biotin-tyramide.

(E) Venn diagram showing the numbers of all enriched proteins (left panel) and ubiquitin-related proteins (right panel) in proximity proteomics using APEX2-CSN5 and TurboID-CSN5.

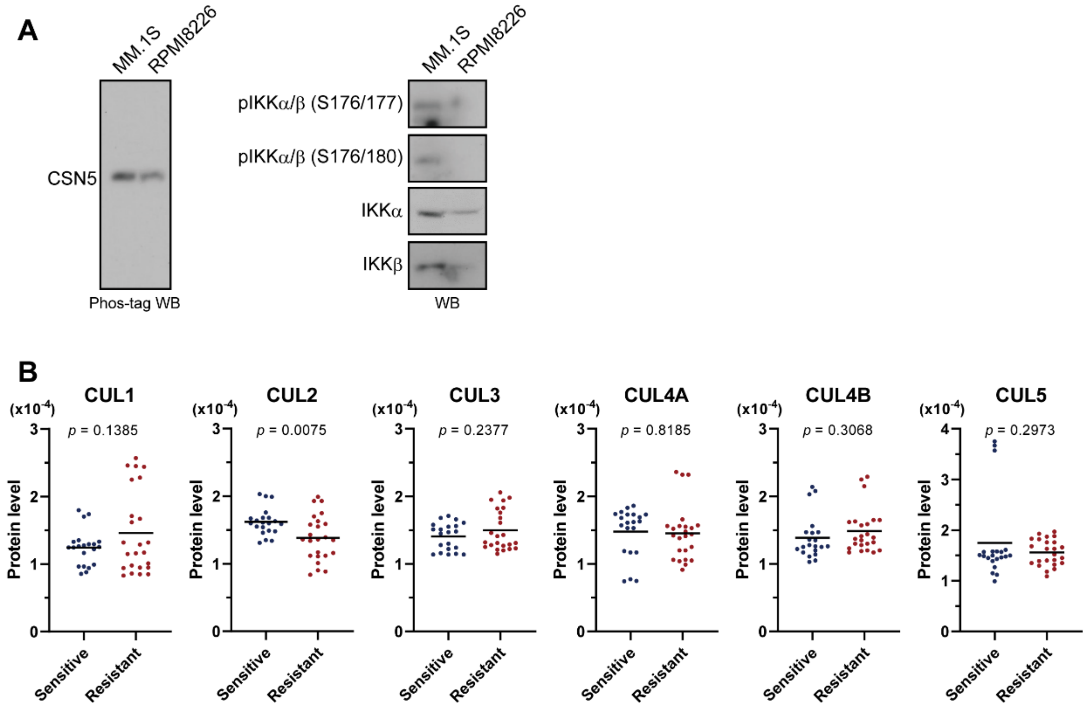

Figure S6 Comparison of candidate proteins involved in the reduction of CSN5 between MM.1S and RPMI8226, related to Figure 6

(A) Western blot combined with Phos-Tag SDS-PAGE were used to compare the phosphorylation state of CSN5 between MM.1S and RPMI8226 cells.

(B) Comparison of the expression levels of CULs identified in proteomics analysis of 15 MM cell lines.
